# Low-dose short infusion ketamine as adjunct to morphine for acute long bone fracture in the emergency department: a randomized controlled trial

**DOI:** 10.1186/s12873-024-00997-w

**Published:** 2024-05-09

**Authors:** Elisa Audrey Eddie, Ahmad Zulkarnain Bin Ahmad Zahedi, Sabariah Faizah Binti Jamaluddin, Julina Md Noor

**Affiliations:** 1 Department of Emergency Medicine, Emergency and Trauma Department, Hospital Tawau, 91007 Tawau, Sabah Malaysia; 2 Department of Emergency Medicine, Faculty of Medicine, University of Malaya, 50603 Kuala Lumpur, Wilayah Persekutuan Malaysia; 3 Department of Emergency Medicine, Faculty of Medicine, Universiti Teknologi MARA, Sungai Buloh Campus, Jalan Hospital, 47000 Sungai Buloh, Selangor Malaysia

**Keywords:** Ketamine, Morphine, Analgesia, Emergency Department, Infusion, Long-bone fracture

## Abstract

**Background:**

Ketamine is recognized as an alternative for pain management; however, concerns about emergent adverse reactions have limited its widespread adoption. This study aimed to assess the efficacy of a short infusion of low-dose ketamine (LDK) compared to intravenous morphine (MOR) as adjunctive analgesia for acute long bone fracture pain.

**Methods:**

This single-blinded, randomized controlled trial was conducted in a single emergency department. Patients with acute long bone fractures and numerical rating scale (NRS) pain scores ≥ 6 following an initial dose of intravenous morphine were assigned to receive either a LDK (0.3 mg/kg) over 15 min or intravenous MOR at a dose of 0.1 mg/kg administered over 5 min. Throughout a 120-min observation period, patients were regularly evaluated for pain level (0–10), side effects, and the need for additional rescue analgesia.

**Results:**

A total of 58 subjects participated, with 27 in the MOR group and 31 in the LDK group. Demographic variables and baseline NRS scores were comparable between the MOR (8.3 ± 1.3) and LDK (8.9 ± 1.2) groups. At 30 min, the LDK group showed a significantly greater mean reduction in NRS scores (3.1 ± 2.03) compared to the MOR group (1.8 ± 1.59) (*p* = 0.009). Similarly, at 60 min, there were significant differences in mean NRS score reductions (LDK 3.5 ± 2.17; MOR mean reduction = 2.4, ± 1.84) with a *p*-value of 0.04. No significant differences were observed at other time intervals. The incidence of dizziness was higher in the LDK group at 19.4% (*p* = 0.026).

**Conclusion:**

Short infusion low-dose ketamine, as an adjunct to morphine, is effective in reducing pain during the initial 30 to 60 min and demonstrated comparability to intravenous morphine alone in reducing pain over the subsequent 60 min for acute long bone fractures. However, it was associated with a higher incidence of dizziness.

**Trial registration:**

NMRR17318438970 (2 May 2018;www.nmrr.gov.my).

## Background

Pain is a common reason for patients to visit the emergency department (ED), and 80% of patients with musculoskeletal injuries experience moderate to severe pain [1]. However, it has been reported that pain control in the ED is frequently suboptimal, even in patients with acute long bone fractures [2–4], which are common causes of severe pain in the ED. Currently, the standard practice for treating acute pain is opioids, but it is not recommended to administer opioids repeatedly to patients with acute pain due to potential adverse effects such as hypotension and respiratory depression [5]. Consequently, there is a need for alternative or adjunct therapies to opioids.

One such alternative medication is ketamine. While ketamine is commonly used in the ED for procedural sedation and as an induction agent for rapid sequence intubation, its use as analgesia has been slower to gain momentum due to reported emergence reactions such as anxiety, nightmares, hallucinations, and delirium [6]. This trend persists in our institution to the present day, despite the increasing recognition of ketamine as a frontline treatment for pain over the past decade in other parts of the world. Recent studies suggest the use of low-dose ketamine for acute pain control. Ketamine at a low dose of 0.1–0.5 mg/kg and particularly at a dose of 0.3 mg/kg has demonstrated an analgesic effect. However, there is a higher incidence of psycho-perceptual adverse effects when ketamine is given as an intravenous push. A recent study by Motov et al. compared the intravenous push of low-dose ketamine versus a short infusion of low-dose ketamine over 15 min for undifferentiated pain in the ED. The study reported that a short infusion of low-dose ketamine significantly reduced unreal adverse effects without compromising analgesic effects [7].

Therefore, the objective of this study is to evaluate the effectiveness of short-infusion low-dose ketamine as an adjunct compared to intravenous morphine alone in managing acute pain resulting from long bone fractures in the emergency department.

## Methods

### Study design

This was a single center, prospective, randomized, single-blinded trial study comparing short infusion low dose ketamine (LDK) versus intravenous morphine as adjunct analgesia for acute long bone fracture pain in ED. The study design and reporting followed the guidelines outlined in the CONSORT statement for randomized controlled trials. The study was approved by Medical Research and Ethics Committee (MREC) Malaysia and was registered with National Medical Research Register NMRR-17–3184-38970. Written and signed informed consent was obtained in accordance with institutional policy.

### Study setting and population

This study was conducted at Hospital Sungai Buloh, Selangor, Malaysia, that annually handles over 150,000 adult ED visits each year. Patient enrolment occurred opportunistically during the study period, dependent on investigator and patient availability. The recruitment period was from May 2018 to February 2019. Before enrolment, ED physicians administered the initial morphine doses to all patients in both study arms following institutional protocols. Numerical Rating Scale (NRS) scores were reassessed after a 15-min interval. Patients with acute long bone fractures received appropriate splints for immobilization.

### Conduct of study

Eligible patients were reassessed and patients with NRS ≥ 6, 15 min after the initial dose of morphine, were recruited into the study. After obtaining written informed consent, each participant enrolled in the study was randomly assigned according to a predetermined randomization list that was generated using IBM SPSS Statistic version 23.0 by the investigator. Participants were randomized to receive either a short infusion of low-dose ketamine (0.3 mg/kg) mixed in 100 ml normal saline solution, administered over 15 min via an infusion pump (Top Infusion Pump Model TOP-2300), or intravenous morphine (0.1 mg/kg) in 10 ml normal saline solution over 5 min via a syringe pump (Top Syringe Pump Model TOP-5300). An ED pharmacist on duty, independent of this study, was notified regarding each patient’s body weight for drug randomization and preparation. The treating staff nurses, who were briefed and trained on the administration of the intervention or control medication before to intervention, administered the medications to eligible patients.

This was a single-blinded protocol study; thus, only the investigator, ED pharmacist, and statisticians possessed knowledge of the study arm to which each patient was randomized. Treating clinicians and patients remained blinded to the study treatment. However, to minimize bias, the researcher appointed independent individuals who were not a part of the study team but trained staff nurses, to assess vital signs and pain scores after administration of study medication. Before initiation of the study protocol, these independent individuals underwent training to evaluate vital signs and pain scores. Consequently, these trained staff nurses were blinded to the treatment arms and recorded pain scores, vital signs, and adverse effects at 30-min intervals after administration of the study treatment. At 30-min intervals, patients were asked about their pain levels and the need for more pain control. They were also evaluated for the presence of side effects and sedation scored according to the Ramsay scale with a threshold set at more than 2 [8]. In instances where a patient required analgesia between scheduled time points, intravenous fentanyl at 1–2 mcg/kg was administered as rescue analgesia, ensuring unhindered access to pain relief without compromising the care provided by clinicians. The total rescue analgesia for both treatment arms was recorded up to 120 min or upon patient admission, whichever occurred first.

Patients eligible for enrolment after they were assessed by attending emergency medicine residents or emergency physicians on duty and meeting specific criteria: long bone fractures of tibia, fibula, femur, radius, ulna, or humerus; 18–60 years old; able to give consent either in Bahasa Malaysia or English; conscious with Glasgow Coma Scale (GCS) score of 15; fracture pain with NRS $\ge 6$ after initial dose of morphine administered by the treating physicians in the ED. Exclusion criteria comprised individuals meeting any of the following conditions: altered mental status (GCS ≤ 14), pregnant, breastfeeding, allergy to ketamine or morphine, hemodynamically unstable vital signs (systolic blood pressure < 90 or > 180 mmHg, pulse rate < 50 or > 150 beats/min, and respiratory rate < 10 or > 30 breaths/min), and a medical history of acute head injury or eye injury, seizure, intracranial bleed, renal or hepatic insufficiency, ischemic heart disease, cerebrovascular accident, asthma or chronic lung disease as well as a history of drug or alcohol abuse, psychiatric illness.

### Outcome

The primary outcomes of this study included the mean reduction in numerical rating scale (NRS) scores from baseline and the mean time required to achieve a reduction of > 3 points in NRS scores. The secondary outcomes encompassed the incidence of adverse events and the mean consumption of rescue analgesia.

### Data analysis

Data were presented as mean ± SD, and proportions were reported as appropriate. Proportions were compared between study groups using the chi-square test. Parametric variables with normal distributions were compared using independent t-tests. Repeated measures were employed to assess changes in pain scores within groups.

We assumed a primary outcome consisting of a minimally clinically meaningful difference of 1.3 between ketamine and morphine groups at the 30-min pain assessment. Assuming an SD of 2.0, a power analysis determined that an independent *t-*test with as sample size of 78 (39 in each group) provided at least 80% power to detect a difference of at least 1.3 at 30 min (and at any other interval postbaseline), with an α = 0.05.

Data entry and analysis were conducted using IBM Statistical Package for Social Science (SPSS) version 23.0 software.

## Results

**Fig. 1:**
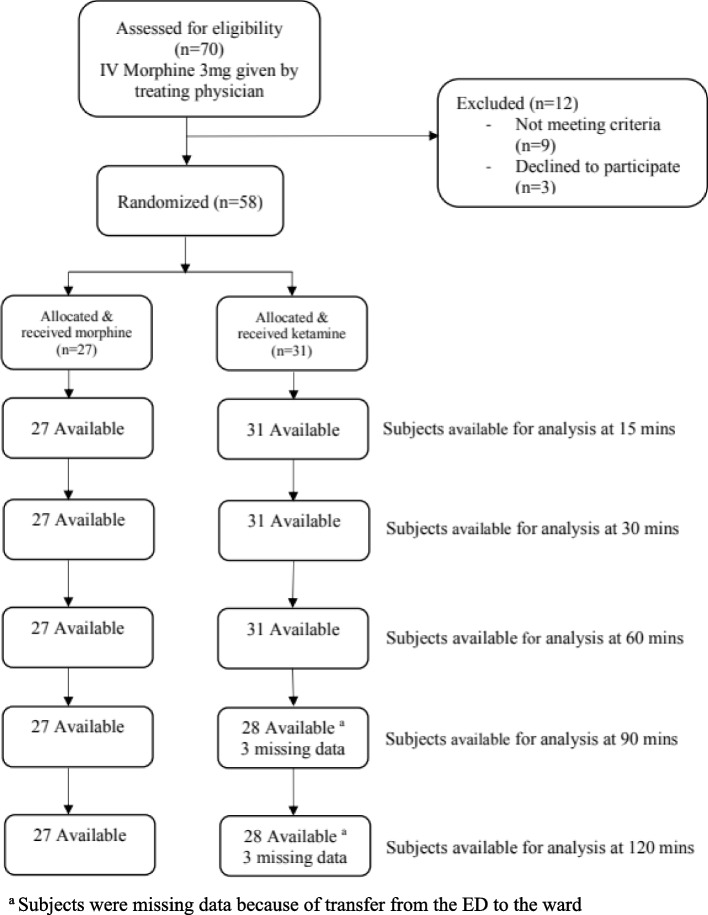
Flow diagram for consented subjects. ^a^Subjects were missing data because of transfer from the ED to the ward

Seventy patients met the eligibility criteria but 12 were excluded (nine due to medical criteria, and three declined participation). Subsequently, 58 patients were enrolled and randomized with 27 to the MOR arm and 31 to the LDK arm (Fig. 1).

Demographic characteristics, including median age, sex, mechanism of injury, and site of fractures, exhibited similarity between the two groups (Table 1). 27 participants in the morphine group and 28 patients in the ketamine group were still observable at 120 min. The most common mechanism of injury was road traffic injury, and the majority of patients sustained lower extremity fractures.

**Table 1:** Baseline patients characteristic

Variable	**Morphine** **(*n* = 27)**	**Low-dose ketamine** **(*n* = 31)**	***P* value**
**Age (years)**	25(12)^a^	27(17)^a^	0.487
**Gender**			
Male (n%)	24(46.2)	28(58.3)	0.861
Female (n%)	3 (50.0)	3 (50.0)	
Weight	64.44(12.3)^b^	66.23(12.7)^b^	0.593
**Mechanism of injury**			
Road Traffic injury (n%)	25(47.2)	28(52.8)	≥ 0.995
Fall (n%)	2(50.0)	2(50.0)	
**Site of fracture**			
Upper extremities (n%)	2 (28.6)	5 (71.4)	0.203
Lower extremities (n%)	26 (50.0)	26 (50.0)	0.90
**Baseline NRS pain score**	8.3 (1.3)^b^	8.9 (1.2)^b^	0.136

^a^Data not normally distributed presented as Median (IQR)

^b^Data presented as mean (SD)

Both treatment groups exhibited reductions in the mean NRS score from baseline, as illustrated in Tables 2 and 3.

**Table 2:** Mean NPRS comparison over time: MOR vs. LDK

Time	IV Morphine Mean ± SD (95% CI)	Low-dose Ketamine Mean ± SD (95% CI)
T0	8.3 ± 1.3 (7.84,8.83)	8.9 ± 1.2 (8.37,9.340)
T15	7.1 ± 0.36 (6.34,7.79)	6.7 ± 0.35 (5.98,7.38)
T30	6.6 ± 0.33 (5.86,7.180)	5.8 ± 0.3 (5.24,6.54)
T60	5.9 ± 0.34 (5.29,6.64)	5.6 ± 0.33 (4.95,6.27)
T90	5.4 ± 0.40 (4.57,6.17)	5.3 ± 0.40 (4.54,6.11)
T120	5.1 ± 0.39 (4.29,5.86)	5.5 ± 0.39 (4.69,6.24)

*SD* Standard Deviation, *CI* Confidence Interval

Repeated Measures ANOVA

**Table 3:** Mean reduction of NRS score from baseline within patient treated between morphine and short infusion low-dose ketamine

Time	Morphine Mean (SD)	Ketamine Mean (SD)	*t*-statistic (d*f*)	Mean difference (95% CI)	*P* value
**T15**^c^	1(2)	2(4)	-1.585^d^		0.113^a^
**T30**	1.8(1.59)	3.1(2.05)	-2.70(56)	-1.3 (-2.29, -0.34)	0.009^b^
**T60**	2.4(1.84)	3.5(2.17)	-2.09(56)	-1.1(-2.27, -0.08)	0.041^b^
**T90**	3.0(2.03)	3.5 (2.63)	-0.90(53)	-0.6 (-1.85, 0.70)	0.371^b^
**T120**	3.3(2.23)	3.4 (2.47)	-0.21 (53)	-0.1 (-1.41, 1.14)	0.834^b^

*SD* Standard Deviation, *CI* Confidence Interval, *df* Degrees of Freedom

^a^Mann-Whitney test

^b^Independent *t* test

^c^Data not normally distributed presented as median (IQR)

^d^Z-statistic

The mean baseline NRS score was ≥ 8 and not significantly different between groups. The mean initial morphine dose in the MOR group was 4.2 mg (SD ± 0.75), and in the LDK group, 4.3 mg (SD ± 0.68). Pain scores in the LDK group, when combined with morphine, exhibited a reduction of more than 3 points at 30 min from baseline (mean reduction = 3.1, SD ± 2.05), in contrast to the MOR group (mean reduction = 1.8, SD ± 1.59), with a *p*-value of 0.009. At 60 min, the mean reductions in NRS score were also significant for the LDK group (mean reduction = 3.5, SD ± 2.17), compared to the MOR group (mean reduction = 2.4, SD ± 1.84), with a *p*-value of 0.041. However, no significant differences were observed at other time intervals.

**Fig. 2:**
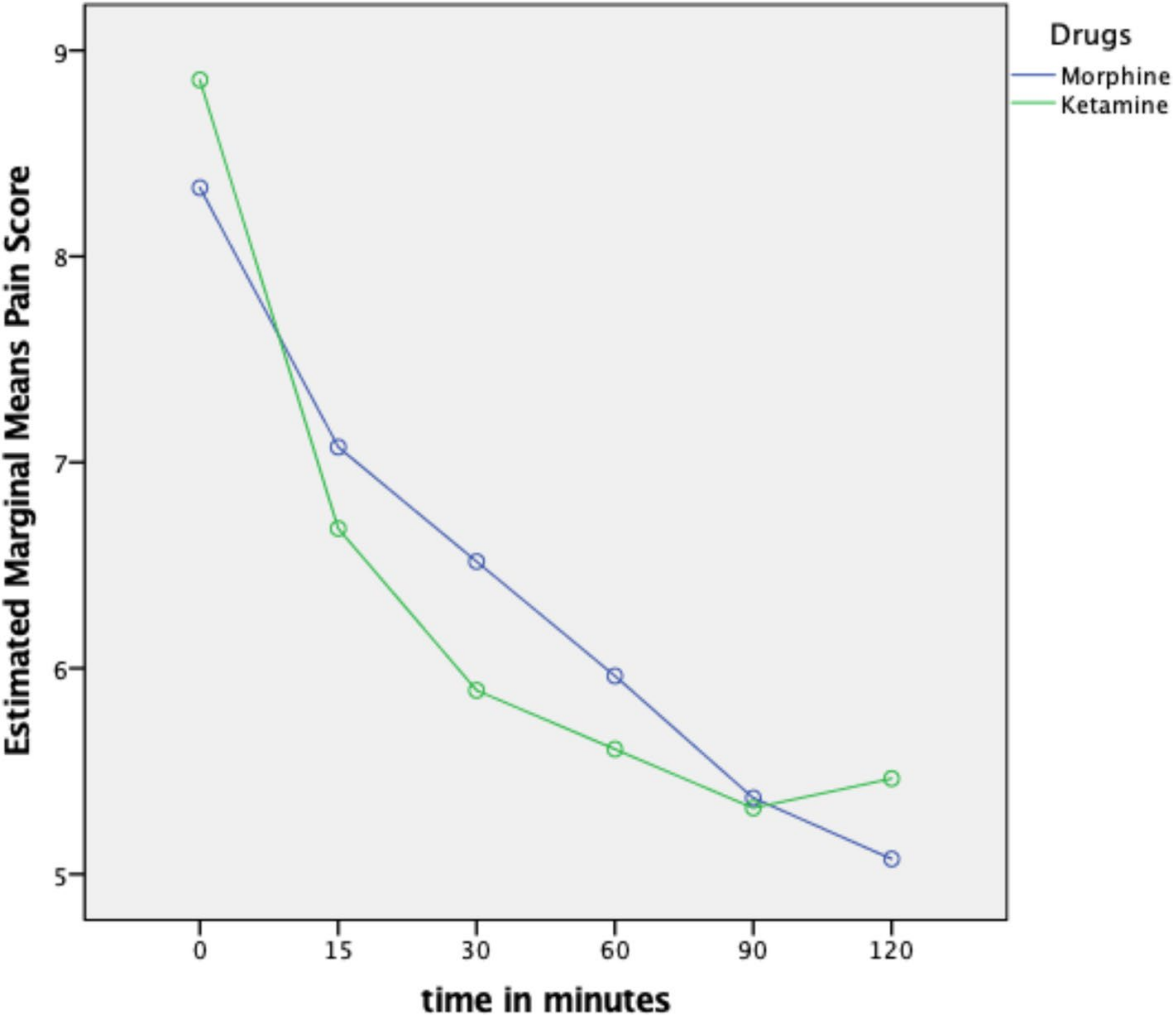
Mean pain score over time

Mean pain scores at each time point for both treatments are depicted in Fig. 2.

The median amount of fentanyl administered as rescue analgesia to both groups was not statistically different (MOR: 50 mcg [IQR ± 8] vs LD: 50 mcg [IQR ± 6]; *p* = 0.921). The numbers of rescue analgesia between both groups were not significantly different (*p* = 0.336) (Table 4).

**Table 4:** Rates of rescue analgesia over time

**Time of rescue analgesia**	**Morphine** ***n* = 27** **n (%)**	**Ketamine** ***n* = 31** **n (%)**	***P* value**^a^
**30 min**^c^	1 (3.7%)^b^	2 (6.4%)^b^	≥ 0.995
**60 min**	4 (14.8%)	3 (9.7%)	0.694
**90 min**	2 (7.4%)	5 (17.9%)	0.432
**120 min**	2 (7.4%)	2 (7.10%)	≥ 0.995
**Total**	9 (33.3%)	10 (32.3%)	0.366

^a^Fisher’s exact test

^b^Frequency (percent)

^c^No rescue analgesia given before 30 min

No serious adverse events were reported in either drug group; these include respiratory distress, seizures, cardiac arrest, or allergic reactions. The prevalence of patient-reported dizziness was higher in the LDK group (19.4%) compared to the MOR group (none), with *p*-value 0.026. Additionally, fatigue and headaches were reported in the LDK group. One patient in the LDK group experienced hallucinations, but no intervention was deemed necessary.

## Discussion

Low-dose ketamine, whether used as an adjunct to opioids or as single agent for analgesia, proves beneficial for acute pain management in ED in adult patients with long bone fracture. This is attributed to its unique mechanism of action blocking the non-competitive antagonist of the *N*-methyl-*D*-aspartic acid (NMDA) receptor and glutamate receptor antagonist. These actions decrease sensitization at the central nervous system and spinal cord levels, resulting in analgesic, hypnotic, and amnestic effects. However, its application as an analgesic in the ED is limited by the adverse effects of emergence reactions [9]. Motov et al. and Miller et al. evaluated ketamine 0.3 mg/kg IV and morphine 0.1 mg/kg IV for acute pain. Both studies measured the change in pain score from baseline to a designated time interval. In Motov et al.'s trial, which included 90 enrolled patients with abdominal pain (71%) or flank pain (17.7%), the mean change in NRS from baseline to 30 min after initial drug administration was not significantly different (0.2 [95% CI -1.19 to 1.46). No serious or life-threatening adverse events occurred, but dizziness (53%) and disorientation (29%) were frequently reported after ketamine injection. In Miller et al.'s study, involving 40 patients at a military hospital, ketamine at 0.3 mg/kg IV and morphine at 0.1 mg/kg IV were administered. However, this study protocol imposed a maximum dose limit of 25 mg for ketamine and 8 mg for morphine.

Despite Motov et al. reporting that the mean NRS from baseline to 30 min between both treatment groups was not statistically significantly different (0.2, 95% CI -1.19 to 1.46) [10], our study demonstrated that means NRS score from baseline to 30 min between treatment groups were statistically significantly different between ketamine and morphine at 30 min and lasting up to 60 min. This may be attributed to the synergic effect of morphine and ketamine, which enhanced the analgesic effect [11]. Johansson et al. demonstrated a significant reduction in the pain score in the morphine-ketamine combination group by 5.4 points in comparison with 3.1 points for morphine alone [12]. Jennings et al. showed that the morphine and ketamine combination is superior to morphine alone with mean pain score change was -5.6 (95% CI -6.2 to -5.0) and -3.2 (95% CI -3.7 to -2.7) respectively [13]. However, the trials by Johansson et al. and Jennings et al. were studied in the prehospital setting with short observation times.

In our study, the use of short infusion low-dose ketamine resulted in a significant decrease in pain score at 30 min after the administration of the drug, which lasted up to 60 min. However, after this interval, no difference in pain score was detected. This finding is contrary to Beaudoin et al., who reported a greater pain reduction in patients who received ketamine over 2 h [14]. Potential explanations for the difference in our findings with those reported by Beaudoin et al. include the amount and timing of rescue analgesia. The median dose of rescue analgesia used by Beaudoin et al. ranged from 5.4—6.1 mg of intravenous morphine. Additionally, the median time at which rescue analgesia was administered ranged between 54–143 min, which may explain the observed reduction of pain score beyond 2 h [14]. In contrast with our study, the median dose of rescue analgesia administered was 50 mcg of intravenous fentanyl, while the mean time rescue analgesia administered ranged between 72–107 min. It could be postulated that an extended infusion period or the administration of repeated doses of ketamine might have resulted in a more sustained reduction in pain scores beyond the initial 60 min. The impact of infusion duration and administration method on the duration of analgesic effects warrants further investigation.

There is a notable agreement between our study and Motov et al.regarding the efficacy of short infusion low-dose ketamine in reducing unreality without compromising the analgesic effects [7]. No serious adverse events were reported during the study, but dizziness was more prevalent in the LDK group compared to the MOR group. These findings were aligned with a previous study conducted by Ahern et al. [15] where 24 of 30 patients (80%) receiving an IV combination of hydromorphone 0.5 mg and ketamine 15 mg experienced dizziness. Beaudoin et al. also reported that 9 of 20 (45%) patients given a combination of IV morphine 0.1 mg/kg and IV ketamine 0.3 mg/kg experienced dizziness [14]. There is a strong probability that the incidence of adverse effects reported in low-dose ketamine may be influenced by the rate of initial bolus administration and the dilution of ketamine. We believe that further trials investigating different ketamine dose ranges and infusion durations may mitigate adverse neuropsychological effects without compromising the analgesic efficacy.

Our work has some limitations. Patient enrolment was subject to investigator availability, potentially introducing bias, and the enrolled subjects may not be entirely representative of the broader population due to stringent inclusion and exclusion criteria. The restricted sample size might have limited the precision of our findings, and a larger cohort could offer more robust insights into potential differences in NRS pain scores.

Furthermore, our research is constrained by its single-blinded nature and the reliance on a single center, which might impact the generalizability of our results. While the investigator was not blinded, the blinding of participants and an independent individual responsible for recording vital signs and pain scores were implemented to mitigate potential biases.

A practical obstacle to the widespread adoption of short infusion low-dose ketamine is the need for an infusion pump, introducing another operational hurdle. However, contemporary infusion pumps, readily available in emergency departments, can be pre-programmed for commonly used medications, including ketamine. This streamlines the process, requiring only the entry of patients' weights, thereby minimizing the risk of drug and dosing pump errors.

In addition, we did not collect data on patient satisfaction and length of ED stay. Including these variables could have provided further insights into the overall patient experience and clinical outcomes, enhancing the comprehensiveness of our findings.

## Conclusion

Short infusion low-dose ketamine, as an adjunct to morphine, effectively reduces pain in the initial 30 to 60 min, demonstrating comparability to intravenous morphine alone in pain reduction over the subsequent 60 min for acute long bone fractures but was associated with a higher incidence of dizziness. This suggests its potential as a viable analgesic option in the emergency department, especially for procedures like splint applications and wound irrigations in long bone fracture patients. Nevertheless, further research is warranted to explore its broader applicability.

## Data Availability

The datas used and/or analysed during the current study are available from the corresponding author on reasonable request.

## Abbreviations

BP: Blood pressure
ED: Emergency department
IBM: International Business Machine
NPRS: Numerical Pain Rating Scale
GCS: Glasgow Coma Scale
IV: Intravenous
LDK: Low-dose short infusion ketamine
MOR: IV morphine
MREC: Medical Research and Ethics Committee
NMDA: *N*-Methyl-*D*-aspartic acid
NMRR: National Medical Research Register
PCA: Patient control analgesia
PSA: Procedural sedation analgesia
SPSS: Statistical Package for the Social Sciences
